# Fescue toxicosis: a detrimental condition that requires a multiapproach solution

**DOI:** 10.1093/af/vfac063

**Published:** 2022-10-14

**Authors:** Gastón F Alfaro, Sonia J Moisá

**Affiliations:** Department of Animal Sciences, Auburn University, Auburn, AL, USA; Department of Animal Sciences, University of Tennessee, Knoxville, TN, USA

**Keywords:** beef cattle performance, blood metabolites, fescue toxicosis, gut microbiome, liver metabolism

**Implications**

- Fescue toxicosis has a broad impact on cattle metabolism and physiology, leading to a reduction in performance and overall profitability.
- The utilization of novel laboratory techniques (i.e., RNA-seq) can reveal the influence of fescue toxicosis in different organs and tissues of animals’ bodies more profoundly.
- Genetic test for fescue toxicosis tolerance could emerge as a predictive technology for improving cattle selection and herd management.

## Introduction

Tall fescue (*Schedonorus arundinaceus* (Schreb.) Dumort.) is a widely used cool-season forage among beef cattle, horses, and sheep farmers worldwide. The popularity of tall fescue relies on the superlative productive characteristics, such as biomass production and nutrient quality in different climatic and edaphic conditions. A clear example of the importance of this pasture in beef production, more specifically in cow-calf operations, is observed in the predominance of tall fescue in spring calving season among producers, in which dams have access to high-quality forage provided by this grass (Kallenbach, 2015). Furthermore, dams not only can utilize tall fescue during the lactation period, but also during the dry period as stockpiled. As discussed, tall fescue represents one of the most used pastures, being present in more than 15 million hectares in the United States alone. However, the superlative aptitudes of tall fescue rely on the symbiotic relationship with an endophyte called *Epichloë coenophiala* (Chai et al., 2020).

The symbiotic relationship between the endophyte and tall fescue plants provides more nutrients to the fungus and greater pest, drought resistance, and yield to the plant. First, regarding herbivory resistance, it has been proposed that different anti-herbivory mechanisms are involved depending on the species consuming the plant. The most important alkaloids with anti-herbivory function in tall fescue can be divided into loline and ergot alkaloids. Loline alkaloids play a role in the anti-herbivory mechanism mainly against insects, acting as metabolic toxins and feeding deterrents, depending on the species. In contrast, ergovaline is toxic mainly to grazing animals. (Bush et al., 1997). Furthermore, the photosynthetic rate of infected plants has also been studied, showing that plants infected with *Epichloë* increase their photosynthetic rate, mainly due to the greater energy required to maintain the plant and the fungus (Rozpądek et al., 2015). Marks and Clay (1996) reported that endophyte-infected (E+) tall fescue plants photosynthesized at a 20% to 25% greater rate than non-infected tall fescue plants. Another benefit of the endophyte infection on the plant is drought resistance. For example, E+ tall fescue presents different physiologic responses to drought than endophyte-free varieties (E−), such as stomatal closure and turgor pressure (Richardson et al., 1993). In addition, since *Epichloë* evolved synergistically with host grasses, there is a specificity among the fungus and cool-season grasses, such as *Epichloë coenophiala* with E+ tall fescue. A possible explanation of the specific synergistic evolution between *Epichloë coenophiala* with E+ tall fescue relies on the asexual reproduction of fungus. Vertical transmission implies a lack of genetic recombination and diversity. The fungus host-specific consociation that improves plant performance is also independent to biotic or abiotic conditions (Lee et al., 2021).

Fescue toxicosis (FT), caused by the consumption of E+ tall fescue by cattle, comprises one of the most important health-related issues in beef production systems worldwide, especially cow-calf operations. For example, FT causes a negative economic impact of approximately $2 billion in the United States annually (Kallenbach, 2015). The fungus produces ergot alkaloids as secondary metabolites, which strategically relocate in the plant based on its reproduction capacity. Since the unique reproduction method of the fungus is asexual, the concentration of the endophyte varies at different locations of the plant (Rottinghaus et al., 1991). Thus, leaves present low ergot alkaloids concentration whereas the crown usually has a medium concentration, and the seed head is the portion of the plant in which the highest level of ergot alkaloids are present (Rottinghaus et al., 1991). The asexual transmission of the endophyte through seeds is an essential strategy for ensuring the survivability of the fungus (Baron and Rigobelo, 2021). Based on this physiological knowledge of the plant, different management strategies have been implemented among producers to avoid the consumption of the plant organs with greater ergot alkaloids concentrations, such as the seed. For example, a rule of thumb among producers is to graze tall fescue at the boot stage. It is possible to increase biomass production by harvesting tall fescue before head emergence or early bloom. However, not only the forage quality decreases during these stages, but more significant risks of FT occurrence take place due to the higher ergot alkaloids concentration in seeds. In addition, implanting consociates pastures (i.e., tall fescue with legumes, such as ladino clover), helps to reduce FT incidences (Villalba et al., 2016). Another advantage of tall fescue-legume consociation is to avoid nitrogen fertilization. It has been previously shown that fertilization with N increases ergot alkaloid content in tall fescue plants (Rottinghaus et al., 1991).

Currently, in order to avoid FT occurrence, tall fescue varieties lacking ergot alkaloids or with beneficial alkaloids are available in the market in some regions, such as the United States, New Zealand, and Europe. Varieties lacking ergot alkaloids are called “endophyte-free tall fescue.” Even though these varieties could help producers decrease the toxicosis occurrence, they are expensive, and the forage aptitude (i.e., biomass production, pest resistance) is usually lower than E+ tall fescue. However, there are also present in the market varieties with endophytes that are beneficial for the plant and avoid the occurrence of FT on animals. These varieties are called “novel endophyte tall fescue”; nevertheless, even though they are superlative in quality compared to “endophyte-free tall fescue,” the utilization of novel endophyte varieties is currently limited due to the cost of implantation.

**Figure 1.**
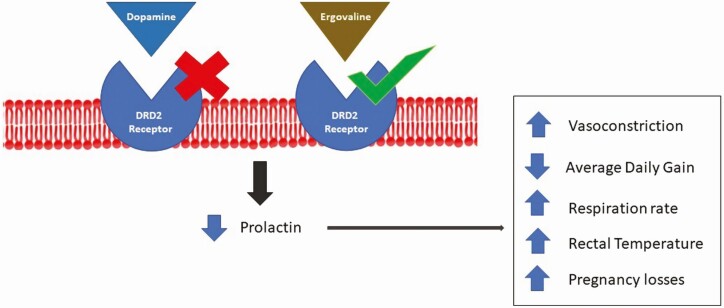
Ergot alkaloids (e.g., ergovaline) influence on dopamine pathway as a causation of FT occurrence.

**Figure 2.**
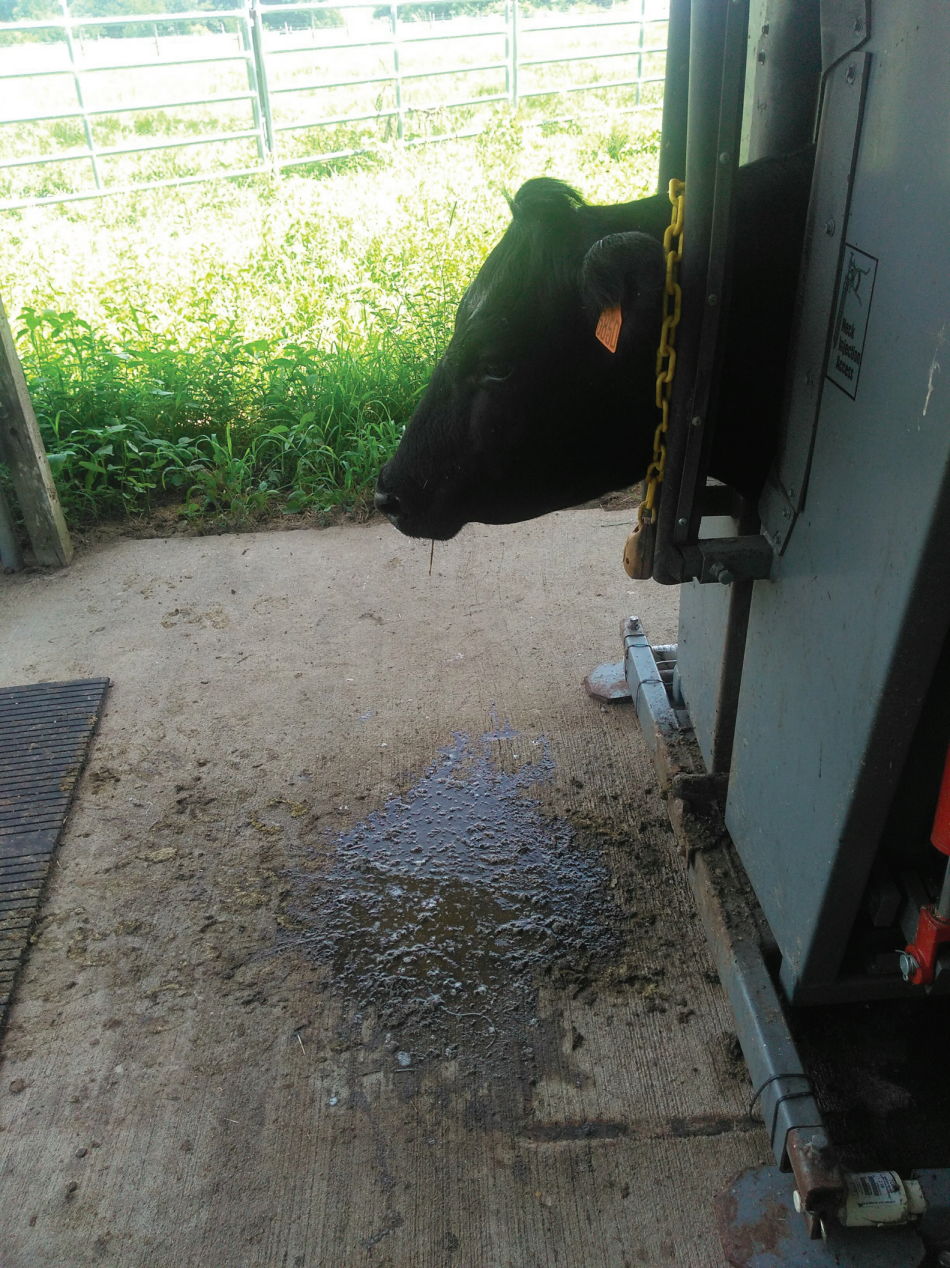
Augmented salivation in animals experiencing FT after 30 d of E+ tall fescue seeds consumption.

Animal performance is negatively affected by the consumption of toxic tall fescue (Figure 1). It has been widely reported that consumption of ergovaline, the most abundant ergot alkaloid in E+ tall fescue, leads to a reduced feed intake, body weight loss, greater rectal temperature and respiration rate, retention of winter coat, excessive salivation (Figure 2) among other detrimental symptoms (Porter and Thompson, 1992; Kallenbach, 2015; Alfaro et al., 2021). Furthermore, ergovaline mimics monoamine neurotransmitters (e.g., dopamine), causing vasoconstriction and an inhibition of prolactin synthesis (Klotz, 2015). Ergovaline is a vasoactive chemical element, and its vasoconstrictive effect affects both peripheral and gastrointestinal vessels, and uterine blood flow. A previous study indicated that a chronic exposure to ergot alkaloids from E+ tall fescue seed reduced ovarian and uterine vessel areas, restricting blood flow to the reproductive organs (Poole et al., 2018). The mechanism of action behind this symptom is also related to the inhibitory effect of ergot alkaloids on amine receptors, in this case, on adrenergic and serotonergic receptors (Klotz, 2015). Since prolactin is a primordial hormone in milk synthesis, one of the most notorious impacts of prolactin synthesis inhibition is the reduction in milk production by lactating beef cows, leading to reduced weaning weights of the offspring (Porter and Thompson, 1992). Several nutritional, managemental, and therapeutic approaches have been proved to reduce the negative impact of FT, showing positive results. From a biological standpoint, ergovaline presents an excellent mechanism of defense against mammal herbivory species due to its unique biochemistry structure that impairs normal animal metabolism by inhibiting amine receptors in the brain, smooth muscle, and internal organs vasculature (Bharadwaj et al., 2020). Based on the evidence discussed, caution must be exercised when E+ tall fescue varieties are included in the forage system planning. Farmers need to have a basic knowledge about the negative effects of grazing E+ tall fescue and the potential management practices for relieving FT (i.e., utilization of E− tall fescue varieties), especially on gestating dams, which leads to offspring’s growth and development impairments. However, the implementation of these practices not always is economically feasible for producers.

The objective of this review is to present, from a multiapproach perspective, the current knowledge on the efforts to dampen the negative impacts of FT on beef cattle production. First, we will describe the current investigation on the effect of ergovaline consumption on the gut microbiome; second, we will present recent evidence of hematological responses to FT; third, we will discuss the relevance of the utilization of genetic test for identifying FT resistance in cattle; and lastly, the impact of FT on liver immunological status from a transcriptome standpoint.

## Gut Microbiome

Recent technological advances in research tools and methodologies have brought new perspectives to the problem of FT. Currently, much attention is focused on ruminal and gut microbiota in different areas of ruminant-related research since it directly affects animals’ physiology, such as immunological status. Even though the ruminal microbial population can degrade ergovaline, the organisms responsible for this action are not yet clearly identified. Therefore, the advantage of utilizing next-generation sequencing analysis in the gut microbiome is to reveal with taxonomic detail the impact of the application of a treatment or the exposure to a detrimental factor, such as E+ tall fescue, in the microbiota population present at the digestive tract level of ruminant species. For example, a recent study conducted by Chai et al. (2020) utilized ewes in gestation and lactation as the animal model for assessing responses to FT. In this study, the authors analyzed the microbial population of rumen samples obtained from ewes exposed to a moderate level and high level of ergot alkaloids concentration in tall fescue. Interestingly, authors found an increment of ruminal microbes involved in toxin degradation at the family-level abundance (i.e., *Coriobacteriaceae* and *Ruminococcaceae*) in ewes exposed to high-endophyte compared with those exposed to medium-endophyte, suggesting that the increase on the abundance of organisms belonging to these families could be a predictor for greater ergovaline degradation. Authors confirmed that the rumen microbiota population is affected by high levels of toxins from E+ tall fescue, which could be associated with host hormone metabolism such as prolactin.

Approximately 30% of the ruminal microbiota is also present in the gut. Based on this foundation and considering that the obtention of ruminal fluid requires the utilization of cannulated animals, which represents an obstacle in the design and execution of research trials, there is a growing, accepted trend of utilizing fecal microbiome as a predictor of the whole ruminal digestive system. A study conducted using Angus steers investigated the impact of E+ tall fescue grazing in gut microbiota diversity. In this study, the authors found that ergovaline intake affected beta diversity; in other words, the microorganisms’ diversity in the gut of animals grazing E+ tall fescue is reduced, potentially decreasing animal productivity due to microbiota perturbations. Interestingly, this study also investigated the effect of heat stress, finding a greater susceptibility on steers exposed to E+ compared with those exposed to E− (Mote et al., 2020). In a feasible future scenario of climate change, which directly affects cattle production and market, it is important to remark that novel investigations aim to recognize and discover the impact of E+ tall fescue under heat stress conditions. Cattle exposed to E+ tall fescue are more prone to experience heat stress mainly due to the occurrence of vasoconstriction and retention of the winter coat (Porter and Thompson, 1992; Alfaro et al., 2021). Consequently, animals’ respiration rate increases during FT as a normal thermoregulatory response for balancing body temperature.

Finally, the literature has reported a correlation between microbiota abundance and physiologic parameters affected by FT. For example, Mote et al. (2019) showed a correlation between fecal microbiota abundance and average daily gain and respiration rate in Angus steers grazing E+. This study indicates that average daily gain is negatively correlated with microorganisms belonging to *Lachnospiraceae* and *Rumiococcaceae* and positively correlated with respiration rate for the same families. In other words, the greater abundance of these microorganisms leads to a reduced average daily gain and a greater respiration rate in animals exposed to E+ compared with those receiving E− diets. A possible explanation of these results is associated with an imbalance in thermogenesis caused by the enteric microbiota affected by FT, that influence and alter lipid availability and absorption (Klotz, 2015; Mote et al., 2020).

## Blood Metabolites

Hematological analyses, such as complete blood count (CBC), have been used extensively by researchers to assess cattle’s health status and generate accurate disease diagnoses (Knowles et al., 2000). The most observed indicator of FT in cattle blood profiles after consumption of E+ tall fescue is a decrease in plasma prolactin levels (Strickland et al., 2011). At the genomic level, genes regulating prolactin production have been targeted to identify genetic markers for tolerance to FT. A single nucleotide polymorphism (SNP) was recently identified within the dopamine receptor D2 (DRD2) gene that was associated with variation in calving rates when grazing E+ tall fescue (Campbell et al., 2014). Furthermore, XK, Kell blood group complex subunit-related family member 4 (XKR4), codifies for a red blood cell membrane protein with a crucial role in facilitating specific recognition of dying cells by phagocytes, and it is associated with serum prolactin levels (Naeini et al., 2020). This is important because as we mentioned before, a reduction in prolactin levels also causes a decrease in milk synthesis, resulting in a lower weaning weight of the offspring.

In our previous study, animals susceptible to FT consuming E+ fescue seeds showed signs of anemia denoted by low mean corpuscular hemoglobin and mean corpuscular volume after 30 d (Alfaro et al., 2021). A previous experiment evaluated the blood components of Angus steers grazing endophyte-infected pastures and observed similar results (Oliver et al., 2000).

In addition, E+ consumption also affects other blood metabolites. For example, Jackson et al. (2015) found reduced circulating glucose in Angus crossbred steers grazing high-endophyte-infected tall fescue compared with low-endophyte tall fescue. The main factor associated with reduced glucose levels can be linked with the decrease in feed intake in animals experiencing FT. However, since ergot alkaloids are detoxified mainly in the rumen, and in the liver, which is the main glucogenic organ in ruminants, the occurrence of FT becomes relevant to assessing glucose levels by disturbances in the liver. Furthermore, albumin, which is the most abundant circulating protein in any adult mammal, is also affected by the intake of E+ diets. For example, a previous study showed that Angus and Angus × Hereford cross steers consuming E+ tall fescue presented lower albumin concentration compared with steers receiving E− diet (Oliver et al., 2000). The reduction in circulating albumin levels can negatively affect blood volume and fatty acids transportation in cattle experiencing FT.

## Genetic Test

**Figure 3.**
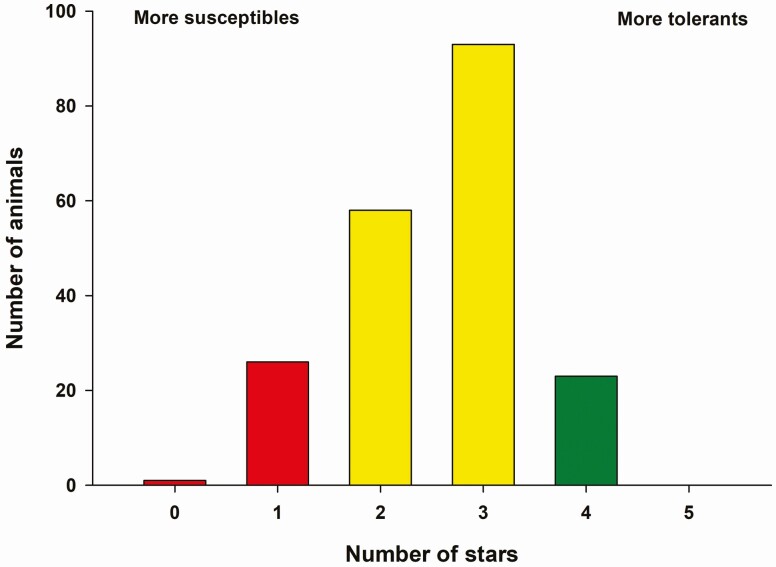
T-snip results obtained from hair root samples in a cowherd of 200 animals.

The selection of animals based on their genetic resistance to FT has been used in commercial beef production systems as a strategy for reducing economic losses caused by this disease. A genetic test developed to determine the level of tolerance or susceptibility to FT (T-snip, Ag. Botanica, Columbia, MO, USA) is currently available to beef producers in the market. This genetic test is under continuous investigation among scientists to fully identify its potential (Galliou et al., 2020). This commercial genetic test provides a tolerance index. The results are usually presented to producers in a six-point rating scale for most susceptible animals (zero stars) or most tolerant animals (five stars; Figure 3). Our research group utilized this genetic test on 200 beef dams, but this genetic test did not provide any clear result that could help to justify its implementation by beef producers to differentiate between tolerant or susceptible animals (Alfaro et al., 2021).

In gestating ewes, vasoconstriction led to a reduction in nutrient partitioning, causing lower performance outcomes in offspring (Porter and Thompson, 1992). We tried to utilize this genetic test on beef cattle to select animals that will overcome with less difficulty the vasoconstrictive effect of ergot alkaloids, like ergovaline present in endophyte-infected tall fescue seeds. Although, when trying to detect differences in vasoconstriction of uterine blood flow in mid-gestation cows and heifers, the genetic test for resistance to FT did not demonstrate any significant effects in uterine blood flow parameters measured using Doppler ultrasonography (Gard Schnuelle et al., 2021).

A critical, current disadvantage of the genetic test is the lack of information regarding the heritability of FT tolerance. Even though selecting dams for FT tolerance can improve the growth and development of the fetus while grazing E+ tall fescue, it is not yet guaranteed that the offspring presents the same level of resistance than the dam and the sire (Galliou et al., 2020). Therefore, testing for genetic resistance to FT may represent a high cost for producers since the testing should be conducted in every generation. Research on this technology is novel and certainly, new insights on heritability will be presented in the literature in the near future.

## Liver Metabolism

The liver is a unique organ responsible for numerous metabolic, vascular, detoxifying, secretory, and excretory functions. Its uniqueness relies on the capability of being regenerated through hepatocyte proliferation after exposure to injury related to a toxin (Michalopoulos, 2007). During the consumption of E+ tall fescue, the liver of different mammalian species experiences a reduction in weight per unit of kg as a result of the numerous detoxification processes and the occurrence of hepatic cell senescence and apoptosis, as shown in rats (Settivari et al., 2006) and beef cattle (Brown et al., 2009). Furthermore, FT downregulates genes related to hepatic energy status. A study conducted by Bhusari et al. (2006) evaluated the effect of FT on hepatic gene expression using mice as the animal model. This study measured mRNA gene expression through microarray and quantitative real-time PCR. There was a significant downregulation for mice fed E+ on genes involved in glycolysis metabolism and that could lead to carbohydrate metabolism impairments due to ergot alkaloids consumption. Similarly, genes that codify for enzymes involved in the synthesis of monounsaturated fatty acids were downregulated in E+ mice. However, FT can also cause upregulation of gene expression related to lipid metabolism. For example, the uptake of high-density lipoprotein (HDL) may be enhanced due to the upregulation of ATP synthase F1 subunit beta (ATP5B) and paraoxonase 1 (PON1), which are associated with homeostasis in lipid circulation levels. Therefore, there is a possible link between the downregulation of genes that codify for enzymes involved in fatty acid synthesis, such as stearoyl-CoA desaturase-1 (SCD1) and the upregulation of ATP5B and PON1, indicating that a lower cholesterol release from the hepatic cell may take place during FT occurrence (Bhusari et al., 2006). These results are partly supported by the findings in a study conducted by Settivari et al. (2006) with the objective of analyzing the effect of FT on hepatic gene expression in male rats, also performing gene expression analyses using microarray and real-time PCR. Authors found a downregulation of the expression of genes related to lipid and carbohydrate status. In addition, FT usually leads to a reduction in average daily gain and body growth. Ergot alkaloids consumption downregulated hepatic body growth-related genes (Settivari et al., 2006).

Finally, ergot alkaloid consumption affects detoxification and antioxidant pathways. Cytochrome P450 (CYP) system, a set of oxidant enzymes generally located at the membrane of hepatocytes, is known for its detoxification capacity. In addition, antioxidant pathways are frequently assessed by measuring the expression of genes that codify for antioxidant enzymes (Strickland et al., 2011). Usually, the expression of genes related to CYP and antioxidant enzymes are targeted as detoxification and redox-status indicators in animals consuming E+ diets. A study performed by Settivari et al. (2008) showed that rats with short-term exposure to ergot alkaloids had an upregulation in hepatic CYP isoforms, whereas genes that codify for antioxidant enzymes, such as superoxide dismutase 1 and 2 (*SOD1* and *SOD2*, respectively) were downregulated. These findings can be supported by similar results obtained in a study conducted by the same authors where an upregulation of CYP isoforms and upregulation of oxidoreductase-related genes were observed (Settivari et al., 2006). Based on this evidence, it is possible to suggest that the pathways affected by FT may lead to animal’s liver to experience oxidative stress and energy metabolism perturbation, resulting in reduced average daily gain and impaired health status.

## Conclusion

Tall fescue still represents an exceptional pasture among cattle producers due to its superlative forage characteristics. Beyond all the efforts to eradicate FT during the last decades, the problem of toxicosis remains present, but fortunately, with a lower negative impact thanks to the continuous discoveries that benefit producers. As reviewed in this article, the approaches to tackle FT are numerous due to the broad impact of this condition on animals’ bodies. Furthermore, we did not discuss in detail the major efforts to dampen FT occurrence focused from the plant side (i.e., E− varieties). Therefore, the positive results obtained from research conducted from the animal and plant standpoints mutually benefit thanks to the synergistic implications of their investigations. The knowledge of the impact of FT on the gut microbiome, blood metabolites, and liver transcriptome may help to develop therapeutic techniques and management procedures that improve animal performance and producer’s profitability.

*Conflict of interest statement*. The authors declare that there is no conflict of interest.

## About the Authors

**Gastón F. Alfaro** is a Post-Doctoral Research Associate at the Panhandle Research and Extension Center of the University of Nebraska-Lincoln, NE. He obtained his MSc and PhD degrees from Auburn University, AL under Moisá’s supervision. His background is associated with beef nutrigenomics with focus on the effects of ruminal-protected nutrients on fetal programming and muscle biology. He is currently working in beef nutrition and management on feedlot operations, in conjunction with extension tasks for improving efficiency and productivity among beef producers in Nebraska.

**Sonia J. Moisá** is a professor in Animal Sciences at the University of Tennessee, Institute of Agriculture (UTIA) in Knoxville, TN. She obtained her MSc and PhD degrees from the University of Illinois at Urbana-Champaign. Her background is in beef nutrigenomics and epigenetics with emphasis in fetal programming. She is currently a member of the UTIA Genomics Center for the Advancement of Agriculture working on develop a program of excellence in livestock genomics with the aim to increase the ability to eliminate genetic defects, accurately predict outcomes, and yield consistent progeny performance. Corresponding author: smoisa@utk.edu
